# Prognostic significance of the red blood cell distribution width in diffuse large B-cell lymphoma patients

**DOI:** 10.18632/oncotarget.16560

**Published:** 2017-03-25

**Authors:** Shujuan Zhou, Fang Fang, Huiyao Chen, Wei Zhang, Yang Chen, Yifen Shi, Zhouyi Zheng, Yongyong Ma, Liyuan Tang, Jianhua Feng, Yu Zhang, Lan Sun, Yi Chen, Bin Liang, Kang Yu, Songfu Jiang

**Affiliations:** ^1^ Department of Hematology, The First Affiliated Hospital of Wenzhou Medical University, Wenzhou, Zhejiang, P.R. China; ^2^ Department of Hematology and oncology, Traditional Chinese Medical Hospital of Zhuji, Shaoxing, Zhejiang, P.R. China

**Keywords:** diffuse large B cell lymphoma, red blood cell distribution width, prognosis, survival

## Abstract

This study examined the prognostic value of the baseline red blood cell distribution width (RDW) in diffuse large B cell lymphoma (DLBCL) patients. The associations between RDW and clinical characteristics were assessed in 161 DLBCL patients from 2005 to 2016. The log-rank test, univariate analysis, and Cox regression analysis were used to evaluate the relationship between RDW and survival. A RDW of 14.1% was considered to be the optimal cut-off value for predicting prognosis. A high RDW was associated with more frequent B symptoms (*P*=0.001), a higher International Prognostic Index score (*P*=0.032), more extranodal sites of disease (*P*=0.035), and significantly lower Eastern Cooperative Oncology Group performance status (*P*=0.031). The log-rank test demonstrated that patients with a high RDW had a shorter overall survival (OS) (2-year OS rate, 53.6% *vs*. 83.6%, *P*<0.001) and progression-free survival (PFS) (2-year PFS rate, 44.7% *vs*. 81.8%, *P*<0.001). The multivariate analysis demonstrated that RDW ≥14.1% was an independent predictor of OS (odds ratio [OR] = 0.345, *P*<0.001) and PFS (OR = 0.393, *P*=0.001). We demonstrated that a high RDW predicted an unfavorable prognosis in patients with DLBCL.

## INTRODUCTION

Diffuse large B-cell lymphoma (DLBCL) is the most common form of lymphoma, accounting for 25–30% of all newly diagnosed cases of adult non-Hodgkin lymphoma (NHL). DLBCL is classified as a heterogeneous entity, encompassing several morphological variants, various biological abnormalities, and variable clinical behaviors and responses to treatment [1]. The International Prognostic Index (IPI), and its variants designed for younger or elderly (*e*.*g*., age-adjusted IPI) patients and patients treated with rituximab (*e*.*g*., revised R-IPI), are the only widely accepted, validated clinical prognostic indices for DLBCL [2, 3]; however, some patients with a favorable IPI fail treatment and vice versa.

Some prognostically significant molecular and immunohistochemical characteristics of DLBCL have been identified, but cost and technical constraints make their routine application impractical; therefore, finding inexpensive, readily available surrogate prognostic markers could make an important contribution to improved risk assessment for individual patients.

Inflammatory cells and soluble mediators, such as cytokines and chemokines, are essential factors that sustain cell growth and invasion, induce angiogenesis, and suppress antitumor immune functions [4, 5]. The presence of systemic inflammation was identified as an independent predictor of the response to treatment, overall survival (OS) and event-free survival in DLBCL patients [6].

The red blood cell distribution width (RDW) is a coefficient of the volume variation of circulating erythrocytes, and is routinely measured in clinical practice as part of a complete blood count (CBC). As an easy-to-measure marker of the systemic inflammatory response, the RDW has been reported in many pathophysiological conditions, including cardiovascular disease and generally increased progressive inflammation [7–11]. The RDW is being increasingly recognized as having an important role in tumor progression and prognosis [12–17].

Here, we present our single-institution experience assessing the prognostic value of the RDW in DLBCL at diagnosis. We retrospectively analyzed a cohort of patients with DLBCL, treated from 2005 to 2016 at our institution, to investigate the prognostic role of RDW at diagnosis in our population in terms of progression-free survival (PFS) and overall survival (OS).

## RESULTS

### Patient characteristics

The analysis included 161 patients [median age = 59 years (range: 18–80 years); 91 (56.5%) males]. The median follow-up time was 42 months (range: 6–120 months).

**Figure 1:**
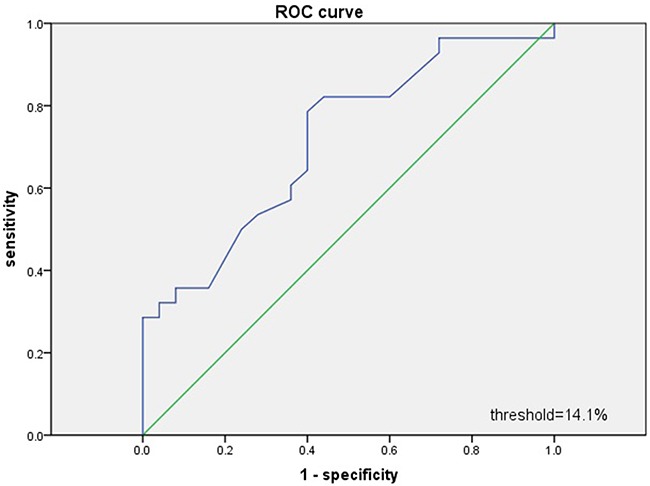
Receiver operating characteristic (ROC) curves analysis for RDW

Based on a receiver operating curve (ROC) analysis of RDW, the patients were divided into low- and high-RDW groups using a value of 14.1%. The area under the curve (AUC) for RDW was 0.716 (95% confidence interval [CI] = 0.578–0.854), and the optimal cutoff value was 14.1%, with 78.6% sensitivity and 64.0% specificity (*P*=0.007; Figure 1). There were 111 patients with a low RDW (< 14.1%) and 50 patients with a high RDW (≥ 14.1%). The patients with a high RDW more frequently showed B symptoms (*P*=0.001) and had a higher IPI (*P*=0.032), more extranodal sites of disease (*P*=0.035), and significantly lower Eastern Cooperative Oncology Group performance status (ECOG-PS) (*P*=0.031). There were no significant correlations between RDW and numerous clinical pathological factors, including age, gender, lactate dehydrogenase (LDH) at diagnosis, disease stage, pathology type, and bone marrow infiltration (Table 1).

**Table 1:** Clinical characteristics of DLBCL patients

Characteristics	Total (n=161)	RDW<14.1 (n=111)	RDW≥14.1 (n=50)	P Value
Gender				
Male, n (%)	91(56.5%)	63(56.8%)	26(52.0%)	0.610
Age	59.1±11.4	58.4±11.0	60.8±12.4	0.206
Ann Arbor stage, n (%)				0.150
I	49(30.4%)	30(27.0%)	19(38.0%)	
II	17(10.6%)	10(9.0%)	7(14.0%)	
III	56(34.8%)	39(35.1%)	17(34.0%)	
IV	39(24.2%)	32(28.8%)	7(14.0%)	
B symptoms, n (%)				0.001
Yes	27(16.8%)	11(9.9%)	16(32.0%)	
No	134(83.2%)	100(90.1%)	34(68.0%)	
ECOG PS, n (%)				0.031
<2	129(80.1%)	94(84.6%)	35(70.0%)	
≥2	32(19.9%)	17(15.4%)	15(30.0%)	
Extranodal sites of disease, n (%)				0.035
>1	32(19.9%)	17(%)	15(30.0%)	
≤1	129(80.1%)	94(%)	35(70.0%)	
IPI, n (%)				0.032
0	33(20.5%)	26(23.4%)	7(14.0%)	
1	60(37.3%)	43(38.7%)	17(34.0%)	
2	32(19.9%)	24(21.6%)	8(16.0%)	
3	23(14.3%)	10(9.0%)	13(26.0%)	
4	10(6.2%)	5(4.5%)	5(10.0%)	
5	3(1.9%)	3(2.7%)	0(0%)	
LDH, n (%)				0.082
≤1 × ULN	100(62.1%)	74(66.7%)	26(52.0%)	
>1 × ULN	61(37.9%)	37(33.3%)	24(48.0%)	
Bone marrow involvement, n (%)				0.287
YES	10(6.2%)	5(4.5%)	5(10.0%)	
NO	151(93.8%)	106(95.5%)	45(90.0%)	
Pathology type				0.843
GCB subtype	39(24.2%)	26(23.4%)	13(26%)	
Non-GCB subtype	122(75.8%)	85(76.6%)	37(74%)	
Hemoglobin(g/L), mean±SD	122.9±19.0	127.8±16.7	112.1±19.4	<0.001
RDW, mean±SD	13.6±1.3	13.0±0.7	15.0±1.2	<0.001

Abbreviations: ECOG, Eastern Cooperative Oncology Group; IPI, International Prognostic Index; LDH, lactate dehydrogenase; PS, performance status; GCB, germinal center B cell; RDW, red blood cell distribution width.

### Association between RDW level and clinical outcome

**Figure 2:**
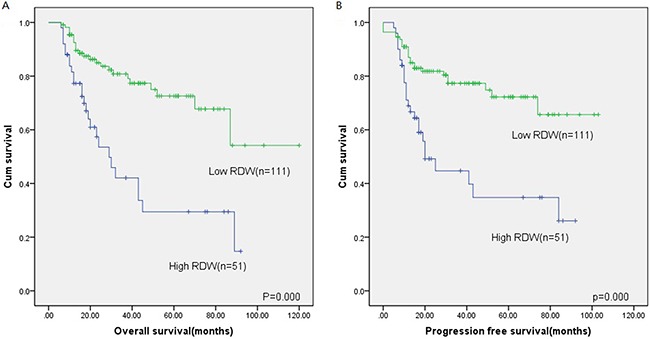
Kaplan-Meier survival analysis of RDW Overall survival **(A)** and progression-free survival **(B)** according to RDW in DLBCL patients.

With a median follow-up of 24 months (range: 6–120 months), patients with a high RDW had a significantly lower PFS than those with a low RDW (2-year PFS, 44.7% *vs*. 81.8%, respectively; *P*=0.000) (Figure 2). The OS showed a similar tendency between the two groups (2-year OS, 53.6% *vs*. 83.6%, *P*=0.000) (Figure 2).

Tables 2 and 3 summarize the results of univariate and multivariate analyses of factors influencing OS and PFS in patients with DLBCL. Univariate Cox regression analysis showed that predictors of OS were a high ECOG-PS (≥ 2, *P*=0.003) and high clinical stage (stages III and IV, *P*=0.016), with B symptoms (*P*=0.002), high IPI (> 2, *P*<0.001), elevated RDW level (*P*<0.001), elevated LDH (*P*=0.003), more extranodal sites of disease (> 1, *P*=0.001), low hemoglobin(*P*<0.001) and bone marrow involvement (*P*=0.026). Independent predictors of PFS were a high ECOG-PS (≥ 2, *P*=0.008), high clinical stage (stages III and IV, *P*=0.024) with B symptoms (*P*=0.001), high IPI (> 2, *P*<0.001), elevated RDW level (*P*<0.001), more extranodal sites of disease (> 1, *P*=0.0003) with bone marrow involvement (*P*=0.049), low hemoglobin(*P*<0.001) and elevated LDH (*P*=0.001) (Table 2)

**Table 2:** Univariate analysis of clinical factors for PFS and OS in 161 patients

Characteristics	OS			PFS		
	OR	95%CI	P value	OR	95%CI	P value
Age(≥60)	1.242	0.711-2.169	0.446	1.243	0.720-2.147	0.435
Gender	1.016	0.580-1.779	0.956	1.020	0.589-1.764	0.944
B symptoms	2.564	1.414-4.649	0.002	2.731	1.510-4.941	0.001
ECOG PS(≥2)	2.525	1.369-4.655	0.003	2.281	1.244-4.184	0.008
LDH(>ULN)	2.333	1.334-4.082	0.003	2.529	1.454-4.396	0.001
Stage (III and IV)	1.994	1.136-3.501	0.016	1.894	1.089-3.293	0.024
Bone marrow involvement	2.656	1.125-6.273	0.026	0.423	0.180-0.997	0.049
IPI(>2)	3.105	1.734-5.559	<0.001	2.901	1.633-5.154	<0.001
RDW≥14.1	3.310	1.890-5.795	<0.001	2.947	1.691-5.137	<0.001
Extranodal sites of disease(>1)	2.724	1.487-4.991	0.001	2.454	1.350-4.458	0.003
Pathology type	1.078	0.537-2.166	0.833	1.193	0.597-2.383	0.618
Hemoglobin	0.973	0.958-0.988	<0.001	0.972	0.958-0.987	<0.001

Abbreviations: ECOG, Eastern Cooperative Oncology Group; PS, performance status; LDH, lactate dehydrogenase; IPI, International Prognostic Index; RDW, red blood cell distribution width.

**Table 3:** Multivariate analysis of clinical factors for PFS and OS

Characteristics	OS			PFS		
	OR	95%CI	P value	OR	95%CI	P value
B symptoms	1.484	0.640-3.444	0.358	1.658	0.733-3.753	0.225
IPI(>2)	1.057	0.389-2.874	0.913	0.974	0.370-2.561	0.957
ECOG PS(≥2)	1.359	0.647-2.855	0.417	1.181	0.583-2.393	0.644
LDH(>ULN)	1.721	0.760-3.898	0.193	2.061	0.929-4.573	0.075
Stage (III and IV)	0.645	0.257-1.616	0.349	0.643	0.265-1.559	0.328
Bone marrow involvement	1.145	0.403-3.251	0.799	1.211	0.434-3.377	0.715
Extranodal sites of disease(>1)	2.561	1.030-6.370	0.053	2.299	0.950-5.565	0.065
RDW ≥ 14.1	3.062	1.669-5.619	<0.001	2.650	1.448-4.849	0.002
Hemoglobin	0.984	0.964-1.004	0.108	0.985	0.966-1.004	0.113

Abbreviations: ECOG, Eastern Cooperative Oncology Group; PS, performance status; LDH, lactate dehydrogenase; IPI, International Prognostic Index; RDW, red blood cell distribution width.

Multivariate analysis, which included all of the parameters significant at *P*<0.05 in the univariate analyses, revealed that RDW ≥ 14.1% was the only independent predictor of OS (odds ratio [OR] = 3.062, 95% CI =1.669–5.619, *P*<0.001) and PFS (OR =2.650, 95% CI = 1.448–4.849, *P*=0.002) (Table 3).

## DISCUSSION

The RDW value, obtained at diagnosis as part of a standard automated CBC, is a novel, immediate prognosticator in DLBCL patients. Our results for DLBCL confirm reports by other investigators [12]; we provide evidence that a high RDW at diagnosis is strongly associated with high-risk clinical features in patients who receive rituximab-based chemotherapy.

RDW is an automated measure of the heterogeneity of red blood cell dimensions (*e*.*g*., anisocytosis) and is performed routinely as part of a CBC. Traditionally, it has played a role in the differential diagnosis of anemia [18]. Recently, RDW is increasingly being recognized to play important roles in carcinogenesis and tumor progression [12, 13, 16, 17]. There is evidence of its prognostic value in various malignancies [15, 19–22]. Some studies and a meta-analysis demonstrated that RDW is a potent predictor of all-cause mortality, including cancer-related deaths [23–25]. In patients with symptomatic multiple myeloma, elevated RDW values were associated with a higher disease stage according to the International Staging System, and a poor prognosis [26]. RDW is reported to be a useful biomarker for distinguishing between benign and malignant breast tumors. An elevated pretreatment RDW may be associated with a worse prognosis in young women with breast cancer [27]. Moreover, RDW elevation is significantly correlated with larger primary tumors, more infiltrated axillary lymph nodes, and advanced stages [15].

The mechanism underlying the relationship between RDW and survival or disease activity is not clear. Research has found an association between RDW and a variety of inflammatory markers, such as high-sensitivity C-reactive protein, the erythrocyte sedimentation rate, interleukin-6, soluble transferrin receptor, and soluble tumor necrosis factor receptors I and II [9]. A high RDW reflects underlying inflammation that impairs erythrocyte maturation and leads to inadequate production of the hormone erythropoietin, undernutrition (*i*.*e*., deficiencies in nutrients such as iron, vitamin B_12_, and folate), oxidative damage, and age-associated diseases via changes in erythropoiesis [28]. Inflammation impairs erythropoiesis and contributes to the increase in RDW. Furthermore, inflammation can cause changes in red blood cell maturation by altering the red cell membrane, leading to increased RDW [29]. It is also associated with impaired iron release from reticuloendothelial macrophages, which can be observed in anemia caused by inflammatory conditions [30].

The role of inflammation in the development of lymphoma has long been recognized and investigated extensively. DLBCL development and invasion depend on multiple interactions between tumor cells and non-neoplastic cells, and on their interaction with the surrounding stroma/matrix environment [31]. In this study, we found a positive association between RDW and B symptoms, a higher IPI, and a lower ECOG- PS. This might also reflect an association between RDW and the increased inflammation or malnutrition caused by cancer progression.

This study was limited in that it was conducted at a single center and included a retrospective analysis of a small number of patients. Further multicenter, prospective studies containing more patients are needed. Despite these limitations, our study suggested that pretreatment RDW is associated with PFS and OS in DLBCL patients treated with rituximab and CHOP (cyclophosphamide, doxorubicin, vincristine, prednisone; R-CHOP) or similar chemotherapy. A high RDW before treatment initiation was an predictor of unfavorable prognosis in DLBCL patients. Based on our findings, we recommend that RDW be used as an easily determined, inexpensive biomarker for risk assessment in patients with DLBCL.

## MATERIALS AND METHODS

### Patients and methods

The inclusion criteria were a diagnosis of *de novo* DLBCL, treatment with R-CHOP or R-CHOP-like chemotherapy (*e*.*g*., EPOCH and CHOEP regimens, used when patients are young, with an elevated LDH and bulky mass, and are of Stage III/ IV; no patients received radiotherapy) for at least four cycles, complete clinical data, and followed at the First Affiliated Hospital of Wenzhou Medical University from 2005 to 2016. The dose of rituximab was 375 mg/m^2^ for all patients. We excluded patients with primary central nervous system lymphoma, transformed NHL, or human immunodeficiency virus-associated DLBCL, and those who were lost to follow-up. Patients with inflammatory conditions, including infections or collagen diseases, anemia, and other diseases of the hematological system, cardiovascular and cerebrovascular disorders, a previous malignancy, pre-treatment with induction chemotherapy or radiotherapy, or non-cancer-associated death, were excluded. From June 2005 until February 2016, 161 patients with DLBCL qualified for the study, which was approved by the Institutional Review Board of the First Affiliated Hospital of Wenzhou Medical University and was performed in accordance with the principles of the Declaration of Helsinki.

The RDW was calculated in routine blood tests performed immediately after DLBCL was diagnosed and before initiating any treatment (pretreatment RDW). Receiver operating characteristic (ROC) curve analysis was used to determine the optimal RDW cutoff. The binary clinical outcome (death/survival) was determined 2 years after diagnosis. Patients were categorized as “alive/censored” when the follow-up time was longer than 2 years, and as “dead” when they died before this time.

The following demographic characteristics, clinical features, and laboratory parameters were obtained from medical records: sex, age, disease stage, IPI, presence of B symptoms, LDH, hemoglobin (Hb), pathology type, ECOG-PS, and number of extranodal locations involved.

The response and relapse criteria were as defined by Cheson *et al*. [32]. OS was defined as the time from diagnosis to death. PFS was defined as the time from diagnosis to relapse.

### Statistical analysis

The statistical analyses were performed using SPSS software (ver. 17.0). Correlations between RDW and clinical parameters were evaluated using the chi-square or Fisher's exact test. OS and PFS were analyzed using Kaplan–Meier curves, which were compared using the log-rank test. Categorical variables were compared using the chi-square test. Variables that were significant at *P*<0.05 in the univariate Cox regression analysis were included in the multivariate analysis using forward stepwise selection. *P*<0.05 was considered statistically significant and all *P*-values were two-tailed.
